# A Machine Learning Model for Detection of Coronary Artery Disease Using Noninvasive Clinical Parameters

**DOI:** 10.3390/life12111933

**Published:** 2022-11-19

**Authors:** Mohammadjavad Sayadi, Vijayakumar Varadarajan, Farahnaz Sadoughi, Sara Chopannejad, Mostafa Langarizadeh

**Affiliations:** 1 Department of Health Information Management, School of Health Management and Information Sciences, Iran University of Medical Sciences, Tehran 14496-14535, Iran; 2 Department of Computer Engineering, Technical and Vocational University (TVU), Tehran 14357-61137, Iran; 3 School of Computer Science and Engineering, The University of New South Wales, Sydney 2052, Australia; 4 Dean International, Ajeenkya D Y Patil University, Pune 412105, India; 5 Swiss School of Business and Management, 1213 Geneva, Switzerland

**Keywords:** coronary artery disease, early detection, machine learning, noninvasive clinical parameters

## Abstract

**Background and Objective:** Coronary artery disease (CAD) is one of the most prevalent causes of death worldwide. The early diagnosis and timely medical care of cardiovascular patients can greatly prevent death and reduce the cost of treatments associated with CAD. In this study, we attempt to prepare a new model for early CAD diagnosis. The proposed model can diagnose CAD based on clinical data and without the use of an invasive procedure. **Methods:** In this paper, machine-learning (ML) techniques were used for the early detection of CAD, which were applied to a CAD dataset known as Z-Alizadeh Sani. Since this dataset has 54 features, the Pearson correlation feature selection method was conducted to identify the most effective features. Then, six machine learning techniques including decision tree, deep learning, logistic regression, random forest, support vector machine (SVM), and Xgboost were employed based on a semi-random-partitioning framework. **Result:** Applying Pearson feature selection to the dataset demonstrated that only eight features were the most effective for CAD diagnosis. The results of running the six machine-learning models on the selected features showed that logistic regression and SVM had the same performance with 95.45% accuracy, 95.91% sensitivity, 91.66% specificity, and a 96.90% F1 score. In addition, the ROC curve indicates a similar result regarding the AUC (0.98). **Conclusions:** Prediction is an important component of medical decision support systems. The results of the present study showed that feature selection has a high impact on machine-learning performance and, regardless of the evaluation metrics of the machine-learning models, determining the effective features is very important. However, SVM and Logistic Regression were designated as the best models according to our selected features.

## 1. Introduction

The world health organization (WHO) has declared that cardiovascular disease (CAD), especially ischemic heart diseases (IHD), is the most common cause of premature death around the world, with 17.9 million deaths annually, equivalent to 31% of all deaths worldwide [1]. CAD is predicted to cause over 23 million deaths for about 30.5% of the world population by 2030 [2]. CAD may be the major cause of abnormal heart rhythms, which may occur suddenly and lead to mortality [3]. IHD killed 8.9 million people worldwide in 2017 and it is currently estimated that 153.5 million people suffer from these diseases [4].The cost of heart diseases in the united states alone is more than USD 200 billion annually and it has been predicted to increase twofold by 2030 [5]. CAD is the most common type of IHD, which occurs when at least one of the coronary arteries has more than 50% stenosis [6]. The early diagnosis and timely medical care of cardiovascular patients can greatly prevent the sudden death of patients and reduce the high costs of surgery and other treatments [7]. Therefore, providing accurate diagnostic and preventive methods can have a significant effect on reducing the death rate of such diseases [8]. A CAD diagnosis is a complex clinical process that requires skilled and experienced physicians, spending a great deal of time and money, using a variety of types of equipment, and obtaining the right result by the investigation of different risk factors such as laboratory tests and physical examinations [9]. The gold standard for CAD diagnosis is invasive coronary angiography [10]. This method is expensive and has various complications; thus, researchers are constantly searching for non-invasive, economical, fast, and valid techniques for early CAD diagnosis [11,12]. Machine learning (ML) can effectively show hidden data relationships and, thus, has been used to establish non-invasive evaluation methods for the diagnosis of various diseases, especially CAD [13]. Data mining has various stages, including data collection, data preprocessing (data preparation), model selection (machine learning algorithms), the training and evaluation of selected model, parameter adjustment, and finally prediction. Machine-learning algorithms are used in many areas such as big data, social networks, the internet of things (IOT), and computer-assisted diagnosis systems [14,15]. However, more research is needed to generalize ML models [16].

Various systems have been proposed for diagnosing CAD using machine learning. Considering that machine-learning models must be used as the basis of a decision support system, it is necessary to first identify the parameters that are effective in diagnosing CAD using statistical tests and experts’ approval, and then implementing the appropriate algorithm for modeling. In other studies, a large number of parameters are used in diagnosis, and it is very difficult for a medical doctor to consider them all. In this article, we tried to identify the minimum number of effective parameters to help physicians and to use the best machine-learning algorithm according to the selected features.

In the next sections, firstly, an explanation of the dataset is presented and then the method and computational environment is explained. Finally, the results are reported, and, in the discussion section, the results are compared with similar studies.

## 2. Dataset

In this study, we used the Z-Alizadeh Sani dataset, which contains 54 features and 303 records of patients at the cardiovascular center of Shahid Rajaei hospital, Tehran. This dataset has two main classes: CAD (216 cases) and normal (87 cases) [17]. The main features of this dataset are four categories: (1) Demographic, (2) symptoms and examination, (3) electrocardiogram, and (4) laboratory and echo features (Table 1).

This dataset is publicly available in the UCI Machine Learning repository for researchers [18]. The main advantage of this dataset is its completeness. There are no missing values or outliers in this dataset and the samples were gathered under Dr. Zahra Alizadeh Sani’s supervision.

## 3. Method

### 3.1. Feature Selection

CAD data include many features, such as demographic indices, symptoms and examination indices, ECG indices, and laboratory and echo indices. Meanwhile, there are irrelevant and unnecessary features that not only increase computational complexity but also decrease a model’s learning accuracy and efficiency [19]. Noisy features and dependent relationships in the heart disease dataset can affect the diagnosis process. As a result, it is necessary to reduce the dimensions of the main dataset via a feature selection method [20]. Feature selection (FS) refers to the process of selecting a subset of the most suitable features based on the real set without considering irrelevant or redundant features [21]. In this study, we used weights that calculated by Relief-f, SVM, and Pearson correlation algorithms [22].

#### 3.1.1. Relief-F

Relief-f algorithm selects top-ranking features from the dataset by assigning different weights to each feature in comparison to its neighbors [23]. In this algorithm, sample-based feature selection method and chi-square test are used as statistical methods. Relief algorithm is concerned with evaluating features based on the similarity of neighbor samples in the analyzed set of samples [24]. This algorithm detects features for a given set of training samples, sample size, and t-related thresholds that are statistically consistent with the objective task. Relief–F, unlike Relief, is not limited to just two classes and it performs more efficiently and can counteract imperfect data. This is important because missed values of features are highly probable [25].

#### 3.1.2. Weights by SVM

In the weights determined by SVM algorithm, the natural vector coefficients of a linear SVM are used as the feature weights. This operator works not only for two classes but for several classes as well. However, the feature values should be numerical [25]. Weighting scheme of SVM uses F-score to measure feature weights. The higher F-score, the more discriminatory features [26].

#### 3.1.3. Pearson Correlation

Pearson correlation-based feature selection is used to find the best subset of features and is integrated with search strategies. The rank correlation coefficient measures similarity between two features and can be used to evaluate the impact of the relationship between them. The rank correlation statistics include spearman correlation, Kendall correlation, and Kruskal and Goodman coefficients. Spearman correlation measures the relationship between two features using a uniform function. Kendall correlation coefficient measures part of the ranks between two datasets [27].

### 3.2. Performance Measure

Various criteria can be considered to evaluate and compare the performance of machine-learning methods [28]. True Positive (TP) rate is the number of samples correctly classified as positive samples (CAD patients). True Negative (TN) rate is the number of samples correctly classified as negative samples (non-CAD patients). False Positive (FP) rate is the number of negative samples that are incorrectly classified as positive samples. False negative (FN) rate is the number of positive samples that are incorrectly classified as negative samples. Accuracy (ACC) (Equation (1)), specificity (Equation (2)), F-1 (Equation (3)), ROC, and AUC (Equation (4)) were the criteria we used to evaluate the efficiency of the algorithms. Accuracy consists of two performance measurement criteria for the classification of models; these criteria have been used widely in other studies, and the F1 measure covers both of them. Accuracy is the ability of each classification to measure the scale of correctly classified samples in all samples [18,19,28,29,30]. The ROC curve is a simple and good criterion that is formed from combinations of the probability of the relative frequencies of correct and incorrect decisions. The ROC curve is plotted based on sensitivity and specificity. A larger area under the curve shows higher diagnosis accuracy. In the ROC, the point near the top left corner of the coordinate graph is the critical value with high sensitivity [31]. The AUC is another important evaluation criterion for the classification of the area under the ROC curve, which is calculated based on true positive rate and false positive rate [29].

ACCURACY = (TP + TN)/(TP + TN + FP + FN)(1)

SPECIFICITY = (2 × TN)/(TN + FP)(2)

F-1 = (2 × TP)/(2 × TP + FP + FN)(3)

(4) $$\mathrm{AUC}=\int_{0}^{1}\frac{\mathrm{TP}}{P}\;d\;\frac{\mathrm{FP}}{N}=\frac{1}{P\cdot N}\int_{0}^{1}\;\mathrm{TPDFP}$$

### 3.3. Modeling and Computational Environment

The Z-Alizadeh Sani dataset contains 71% positive records (patient with CAD) and 29% negative records (normal patients). Asus Z-Book S13 computer equipped with Intel 2 GHZ CPU core i7 and 16 GB RAM and employing the Ubuntu Linux 19.04 operating system were used for simulation. The models developed using Python programming language version 3.8.1. Pandas libraries were used to analyze data correlations and Scikit–Learn was used to implement Decision Tree, Logistic Regression, SVM, and Random Forests. Keras is another library that was used to implement the neural network model. All libraries are open source.

Decision Tree, Random Forest, Logistic Regression, Support Vector Machine (SVM), XGboost, and Deep Learning were implemented for modeling, using Cross Validation as partitioning dataset for training set and test set. The performance of each algorithm is evaluated by the criteria mentioned in the previous section and then the best models were selected.

## 4. Results and Discussion

In this section, the results of the mentioned feature selection and machine-learning models are presented.

### 4.1. Results Modeling

Table 2 shows the features that are selected by the feature selection methods. The features selected by each feature selection method are different, so it is necessary to observe the effect of each selected subset on the accuracy of our machine-learning models.

**Figure 1:**
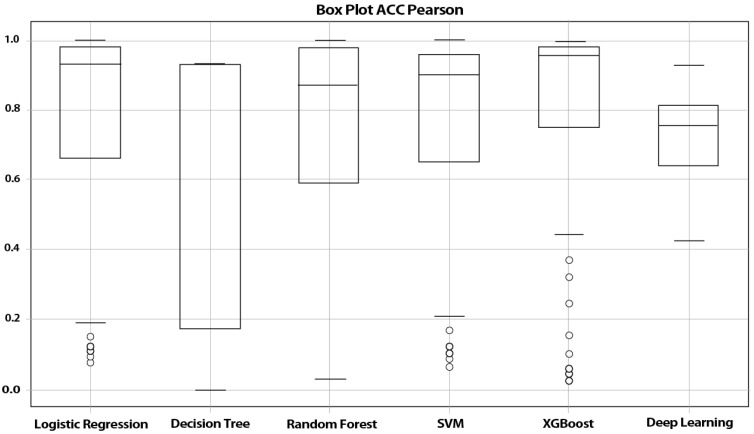
Results of models’ performance based on accuracy.

**Figure 2:**
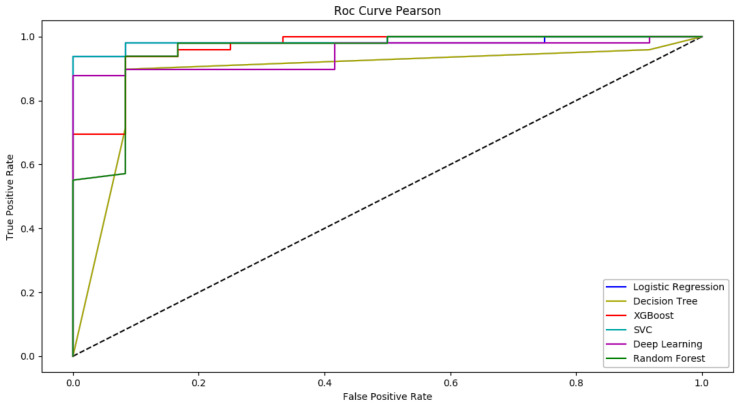
ROC curve diagram.

We used six Machine-Learning models and the accuracy of these models for each feature selection method is shown in Table 3. The data in Table 3 show that the best performance was obtained when we used the Pearson Correlation method as the feature selection method. Therefore, the Pearson Correlation method was selected as our feature selection method. According to the features selected through the Pearson correlation analysis, the selected machine-learning algorithms were tested based on a semi–random [32,33] partitioning framework. The performance evaluation of these models according to their accuracy showed that Logistic Regression and SVM with equal accuracy of 95.8% demonstrated the highest performance. Via a box plot graph, the results regarding the models’ accuracy are presented in Figure 1. Sensitivity (95.91), specificity (91.66), and the F1 score (96.90) are equal in logistic Regression and SVM (Table 4). In addition, the results of the ROC curve investigation as shown in Figure 2 indicate that both SVM and Logistic Regression have almost the same AUC at 0.98.

### 4.2. Performance Comparison of Different Methods

Table 5 shows the comparative results of our proposed method with other machine-learning methods using the Z-Alizadeh Sani data set. The logistic regression model has obtained acceptable results. Our method for CAD diagnosis obtained 95.45% accuracy, 95.91% sensitivity, 91.66% specificity, and an F1 score of 96.90.

### 4.3. Discussion

Due to the advancement of information technology, it is possible to evaluate CAD patients by examining their physicochemical and biochemical features at a lower cost. Although some of the information provided in this area is valuable for CAD diagnosis, there is actually no international standard method for CAD diagnosis [23]. In this study, the Pearson correlation coefficient was used to find the best subset of features among all the features in the Z-Alizadeh Sani dataset; finally, among the various models that we tested on the basis of the features selected from the semi-randomly partitioned data, we determined that the Logistic Regression and SVM models outperformed the other models with 95.45% accuracy, 95.91% sensitivity, 91.66% specificity, an F1 score of 96.90, and an AUC of 0.98. Table 5 compares the results of our proposed method with other studies based on this dataset. As shown in Table 5, our proposed method yields good results in comparison to other studies.

According to Table 3, the best performance for feature selection was achieved by the Person Correlation method. In their study based on the Z- Alizade Sani dataset, Wang et al. developed a two-stage cumulative model with which they, firstly, used the Pearson Correlation coefficient to find the least-correlated classifiers and, secondly, used a counting algorithm to find the best hybrid classifiers; consequently, they achieved 95.43% accuracy, 95.84% sensitivity, and 44.94% specificity [31]. In their study, Jalali et al. also used a feature selection method called correlation-based feature selection to identify the most effective features and suggested that the most appropriate model to classify patients with CAD is the MLP model trained by MVO, as its 96.1% accuracy outperformed the other learning methods under analysis. These studies yielded better results than ours [29].

According to the results presented in Table 5, it can be seen that most of studies have used the 10-fold method to reduce the bias caused by the small and unbalanced dataset. In cross-validation, the ratio of distributed classes can be unbalanced. Therefore, the results of the classification algorithms trained based on this method may be incorrect [19]. In order to avoid bias in this study, a semi-random data-portioning framework was used. In this method, data classification is performed randomly over three stages in each subset, which was used to describe the selected features in detail.

Eventually, the training and test sets for the entire dataset were obtained by integrating all the training and test subsets, respectively. Training the models under a semi-random data-partitioning framework has made this study valuable. According to the performance metrics, Sensitivity and Specificity are high. This is very important in clinical decision support systems because it means more patients can be correctly diagnosed.

The performance of the proposed method is one of the best in the literature and indicates that the selection of the most important features and appropriate training model can significantly improve the performance of machine-learning algorithms. However, the proposed method also had its limitations; for instance, the set of data was small and not well balanced, so to eliminate this limitation, a semi–random data partitioning framework was used.

## 5. Conclusions

The process of disease prediction in the medical sciences is important for clinical decision support systems. This process is facilitated using targeted and rational methods, such as machine-learning and data-mining algorithms. Data-mining techniques can be used to analyze raw data in order to provide new insights into disease diagnosis with accurate predictions. Heart disease is one of the leading causes of death around the world. The diagnosis of this disease as soon as possible is very important to prevent death. Health communities are looking for ways to effectively predict, diagnose, and treat such diseases. The results of the present study showed that using data-mining algorithms such as the SVM model can be useful for predicting CAD. However, further research is needed to compare the performance of different algorithms and determine the best model. In this study, several machine-learning algorithms were implemented on the Alizadeh Sani dataset and the results were discussed. Furthermore, feature selection techniques have also been used to improve the method’s accuracy. Accordingly, applying the proposed method can determine the condition of CAD at a low cost and with high accuracy. The size of a database and the quality of its data are two effective factors in ML. We aim to evaluate the model based on other datasets in the future, establish a larger dataset for CAD by collaborating with hospitals, and develop more vigorous models by extracting more features from physiological signals.

## Figures and Tables

**Table 1:** Features of Alizadeh Sani’s dataset.

Demographic	Age	30–86	Symptom and Examination	PR (Pulse Rate) (ppm)	50–110
	Weight	48–120		Edema	Yes, No
	Sex Male, Female	Male, Female		Weak peripheral pulse	Yes, No
	BMI (Body Mass Index Kg/m^2^)	18–41		Lung Rales	Yes, No
	DM (Diabetes mellitus)	Yes, No		Systolic murmur	Yes, No
	HTN (Hypertension)	Yes, No		Diastolic murmur	Yes, No
	Current Smoker	Yes, No		Typical Chest Pain	Yes, No
	Ex-Smoker	Yes, No		Dyspnea	Yes, No
	FH (Family History)	Yes, No		Function Class	1,2,3,4,
	Obesity	Yes if MBI > 25		Atypical	Yes, No
	No otherwise	No otherwise		Nonanginal CP	Yes, No
	CRF (Chronic Renal Failure)	Yes, No		Exertional CP (Exertional Chest Pain)	Yes, No
	CVA (Cerebrovascular Accident)	Yes, No		Low ThAng (low-Threshold angina)	Yes, No
	Airway Disease	Yes, No	ECG	Rhythm	Sin, AF
	Thyroid Disease	Yes, No		Q Wave	Yes, No
	CHF (Congestive Heart Failure)	Yes, No		ST Elevation	Yes, No
				ST Depression	Yes, No
	DLP (Dyslipidemia)	Yes, No		T inversion	Yes, No
	BP (Blood Pressure: mmHg)	90–190		LVH (Left Ventricular Hypertrophy)	Yes, No
				Poor R Progression (Poor R Wave Progression)	Yes, No
Laboratory and Echo	FBS (mg/dL) (Fasting Blood Sugar)	62–400	Laboratory and Echo	K (Potassium) (mEq/lit)	3.0–6.6
	Cr (creatine) (mg/dL)	0.5–2.2		Na (Sodium) (mEq/lit)	128–156
	TG (Triglyceride) (mg/dL)	37–1050		WBC (White Blood Cell) (cells/mL)	3700–18,000
	LDL (mg/dL) (Low-density lipoprotein)	18–232		Lymph (Lymphocyte) (%)	7–60
	HDL (mg/dL) (High-density lipoprotein)	15–111		Neut (Neutrophil) (%)	32–89
				PLT (Platelet) (1000/mL )	25–742
	BUN (mg/dL) (Blood Urea Nitrogen)	6–52		EF (Ejection Fraction) (%)	15–60
	ESR (mm/h) (Erythrocyte Sedimentation rate)	1–90		Region with RWMA (Regional Wall Motion Abnormality)	0,1,2,3,4
	HB (Hemoglobin) (g/dL)	8.9–17.6		VHD (Valvular Heart Disease)	Normal, Mild, Moderate, Severe

**Table 2:** Features selected by three feature selection methods.

Relief-F	Weights by SVM	Pearson Correlation
CVA Thyroid Disease CHF Diastolic Murmur Typical Chest Pain Atypical Non-anginal LowTHAng BBB (Bundle branch block)	HTN Sex BM DM FH Dyspnea Atypical Tinversion HB LVH Typical Chest Pain Atypical St Elevation Region RWMA	DM FH Atypical Tinversion HB Typical Chest Pain Atypical Region RWMA

**Table 3:** Comparison of algorithms using feature selection method.

Method	Accuracy		
	Relief-F	Weights by SVM	Pearson Correlation
Decision Tree	81/9672	90/1639	90/16
Deep Learning	80/9836	75/7377	79/26
**Logistic Regression**	83/6065	93/4426	**95/08**
Random Forest	81/9672	87/0491	93/44
**SVM (svc)**	83/6065	90/1639	**95/08**
XGboost	83/6065	93/44,262	91/80

**Table 4:** Performance comparison of different methods.

Method	Accuracy	Sensitivity	Specificity	F1
Decision Tree	90/16	89.79	91.66	93.61
Deep Learning	79/26	90.91	31.66	86.21
Logistic Regression	**95/08**	**95.91**	**91.66**	**96.90**
Random Forest	93.44	93.87	91.66	95.83
SVM	**95.08**	**95.91**	**91.66**	**96.90**
Xgboost	91/80	93.87	83.33	94.84

**Table 5:** Comparison of the accuracy of the proposed model with state-of-the-art techniques for the Z-Alizadeh Sani CAD data.

Study	Model	Feature Selection	KF	Accuracy	F1 (%)	Year
Alizadeh sani et al. [18,34,35,36]	SMO	Information Gain Gini Index	10-fold cross validation	84.16	85.55	2013
Alizadeh sani et al. [26]	Genetic + NN	Weight by SVM	10-fold cross validation	93.85	Not reported	2017
Kolukisa et al. [23]	Ensemble Classifier with FLDA	Hybrid feature selection	10-fold cross validation	95	92.74	2019
Ghiasi [37]	CART	-	10-fold cross validation	100	100	2020
Joloudari et al. [38]	RT	Feature-ranking selection method	10-fold cross validation	91.47	Not reported	2020
Jalali [29]	MLP	Correlation-based feature	-	96.1	Not reported	2019
Abdar al. [39,40]	N2Genetic-nuSVM	GA and PSO	10-fold cross validation	93.08	91.51	2019
Wang [31]	Stacking-Based Model	RFECV	10-fold cross validation	95.43	96.77	2020
This study	SVM(svc)—Logistic Regression	Pearson Correlation	Semi-random data partitioning	95.08	96.90	2022

## Data Availability

The dataset is available and accessible via reference [17].
